# Study of non-syndromic thumb aplasia in six independent cases

**DOI:** 10.12669/pjms.303.4626

**Published:** 2014

**Authors:** Hafiza Fizzah Riaz, Karmoon Lal, Bashir Ahmad, Muhammad Shuaib, Syeda Farwa Naqvi, Sajid Malik

**Affiliations:** 1 Hafiza Fizzah Riaz, Human Genetics Program, Department of Animal Sciences, Faculty of Biological Sciences, Quaid-i-Azam University, Islamabad 45320, Pakistan.; 2 Karmoon Lal, Human Genetics Program, Department of Animal Sciences, Faculty of Biological Sciences, Quaid-i-Azam University, Islamabad 45320, Pakistan.; 3 Bashir Ahmad, Human Genetics Program, Department of Animal Sciences, Faculty of Biological Sciences, Quaid-i-Azam University, Islamabad 45320, Pakistan.; 4 Muhammad Shuaib, Human Genetics Program, Department of Animal Sciences, Faculty of Biological Sciences, Quaid-i-Azam University, Islamabad 45320, Pakistan.; 5 Syeda Farwa Naqvi, Human Genetics Program, Department of Animal Sciences, Faculty of Biological Sciences, Quaid-i-Azam University, Islamabad 45320, Pakistan.; 6 Sajid Malik, Human Genetics Program, Department of Animal Sciences, Faculty of Biological Sciences, Quaid-i-Azam University, Islamabad 45320, Pakistan.

**Keywords:** Absence deformity, Absent digit, Oligodactyly, Limb anomaly, Limb defects, Thumb aplasia, Pakistani subjects

## Abstract

***Objectives:*** To report on six independent and isolated cases demonstrating thumb aplasia as an essentially limb-specific phenotype.

***Methods:*** The subjects were ascertained during 2011-2013 from six different geographic regions of Pakistan, and underwent detailed clinical and phenotypic examination.

***Results:*** The affected arms of patients had complete absence of first digital rays, medial inclinations of second and fifth fingers, narrowing of palms, missing carpals, and shortening of zeugopod. All the subjects were presented with isolated and sporadic limb deficiencies, and five had no family history of limb or any other malformation. Parental consanguinity was denied in majority of the cases. We present detailed phenotypic manifestation of thumb apalsia in these subjects.

***Conclusion: ***Thumb aplasia markedly impairs the normal function of affected hand. Surgical procedures like pollicisation of the index finger should be employed to improve the quality of life of these subjects. There is so far no specific genetic factor known for isolated thumb aplasia, compromising an accurate genetic counseling. Collection of patients with similar phenotypic presentations could be useful in further molecular genetic investigations.

## INTRODUCTION

Congenital reduction of thumb is a relatively heterogeneous malformation of first digital ray (OMIM-188100) ^1^^,^^2^ The condition ranges from hypoplastic thumb to a complete absence of first digit and its components.^3^ The variation in the clinical presentation of hypoplastic thumb has lead to the suggestion of their anatomical categorization into five entities which depict increasing severity of thumb deficiency.^4^^,^^5^ Complete reduction of first digit, classified as type V, is a rare malformation and was observed to be 0.25/10,000 births.^6^ It occurs more often in males than in females and is bilateral in about two-third of the cases.^2^^,^^3^^,^^5^ Most of the isolated cases of absent thumb are sporadic. For the familial cases, an autosomal dominant inheritance is witnessed particularly the types associated with other anomalies.^1^^,^^2^ The most common association is radial deficiency of varying degrees.^3^^,^^5^ Thumb agenesis accompanies Holt-Oram syndrome (OMIM-142900), VACTERL association (OMIM-192350), phocomelia, Seckel syndrome (OMIM-210600), thrombocytopenia-absent radius syndrome (OMIM-274000), Fanconi anemia (OMIM-227650), and Duane-radial ray syndrome (OMIM-607323). In majority of these syndromes cardiac, renal, skeletal, ocular and hematological organs are involved.^1^ Mutations in various genes like *FANCE*, *SF3B4*, *WNT7A* and *ATR* have been implicated in Fanconi anemia, acrofacial dysostosis-1, fibular aplasia with poly-,syn-,oligodactyly, and Seckel syndrome-1, respectively, suggesting their likely role in thumb pathomorphogenesis.^1^

Data on limb dysmorphology are scarce for the Pakistani patients.^7^^,^^8^ Here, we report on six different cases with thumb agenesis, presented as a limb-specific phenotype.

## SUBJECTS

 Six patients (4M, 2F) with thumb deficiencies were recruited during 2011-2013 from various geographic regions/medical institutes across Pakistan (Table-I). An informed consent was obtained from each individual or his/her parents. Detail of socio-demographic and biological parameters were obtained. Pedigrees up to three generations were constructed to rule out history of any congenital anomaly and to ascertain the mode of inheritance. Parental marriage types were documented and inbreeding coefficient (*F*) was calculated in order to account for a likely recessive inheritance. Clinical data with respect to the limb were acquired, accordingly and general medical examination involving vital organs was performed. Photographs of affected hands of all subjects and radiographs of two subjects were obtained. Cases were classified according to the revised scheme of hypoplastic thumb by Blauth and Schneider-Sickert^4^ and James *et al*.^5^

The socio-demographic and biological attributes of the recruited cases including parental consanguinity, sibship composition and family history of any congenital malformation, are mentioned in Table-I. The key presentation in all cases was thumb aplasia. Following are the key clinical findings in the recruited patients (summarized in Table-II).

**Fig.1:**
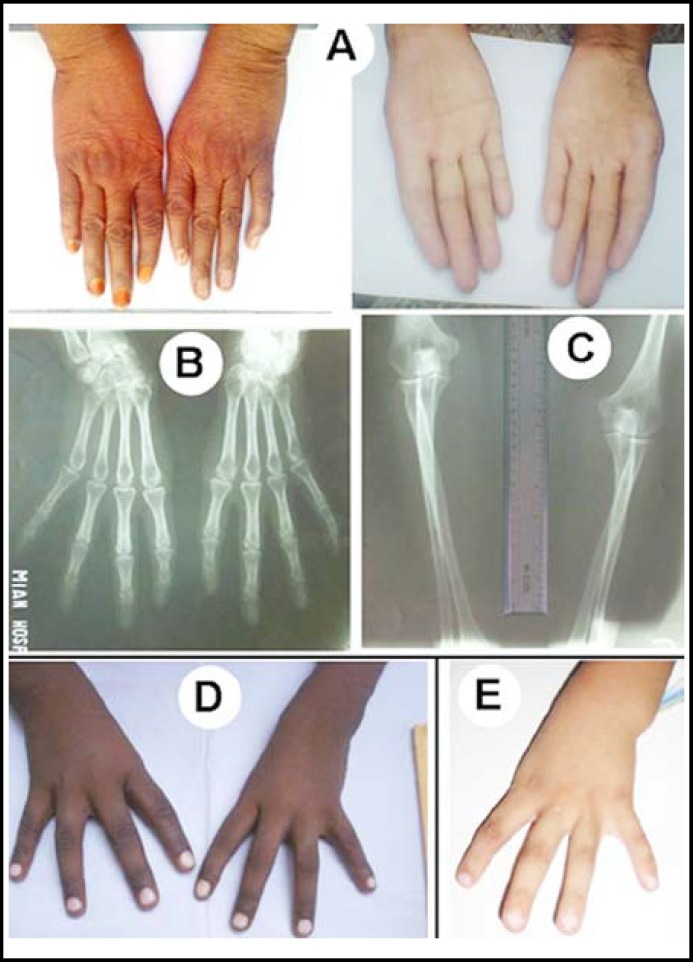
Phenotypic manifestation in patients I—III.

**Case I: **The patient was a 45 year house-wife belonging to a rural area (Table-I). There was parental consanguinity (inbreeding coefficient, *F*=0.0625), and she had five unaffected sibs. She herself had a non-consanguineous union (*F*≤ 0.0156), and had five normal offspring. On physical examination, she was observed to have bilateral and symmetrical aplasia of thumbs (Fig. 1A). Index fingers were medially deviated. There was evidence of clinodactyly in the left hand. Additionally, both arms were slightly short in size (Table-II). The subject was well-adopted to perform her routine house-hold activities. Roentgenographic study revealed that first digital ray along with its components was completely omitted, bilaterally (Fig. 1B). In the right hand, there was a general crowding of carpals; trapezoid was absent and trapezium was hypoplastic. In the left hand, trapezoid and scaphoid were missing while trapezium was dysplastic (Fig. 1B). Along the ulnar-axis, pisiform was not visible. Distal heads of radius and ulna were hypoplastic (Fig. 1C).

**Case II: **This male patient of age 6 originated from a rural area of interior Sindh (Table-I). There was bilateral absence of thumbs and mild shortening of arms (Table-II; Fig. 1D).

**Case III: **The six years school-going male subject belonged to South KhyberPakhtunkhwa (KPK). He was observed to have unilateral aplasia of left thumb (Fig. 1E). Additionally, there was mild medial deviation of index finger and shortening of the arm (Table-II).

**Fig.2:**
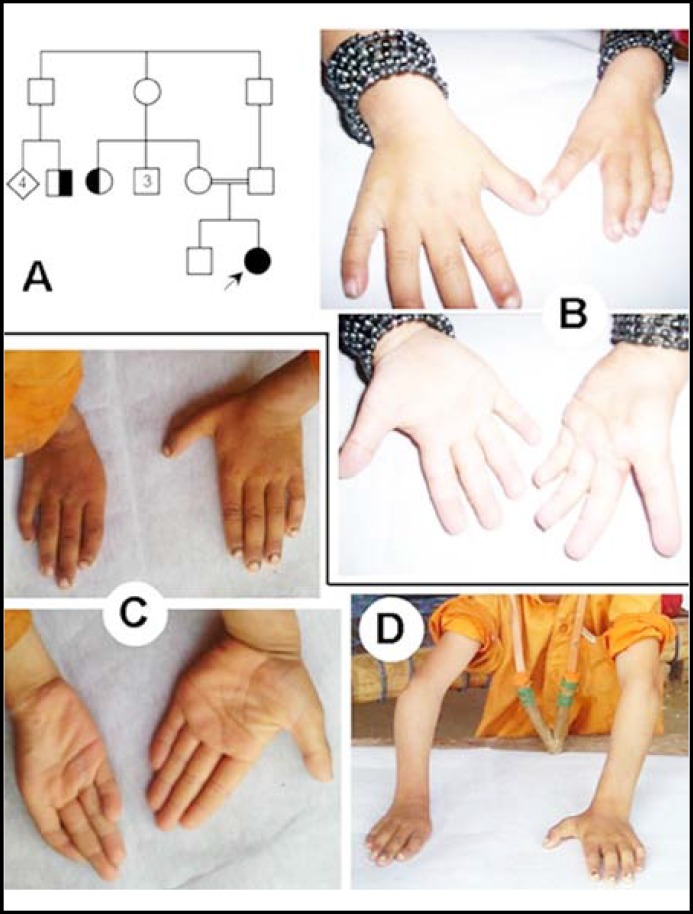
Phenotype in patients IV—V.

**Case IV: **The patient was a three years, otherwise healthy and jolly baby-girl. She was a product of first-cousin union (*F*= 0.0625) (Table-I). There was unilateral aplasia of left thumb (Fig. 2B). There was a history of second/third toes syndactyly in a maternal aunt, and polydactyly (postaxial type B in hands only) in a fourth degree relative (Fig. 2A).

**Case V: **The seven years male subjects originated from North KPK (Table-I). He had short right arm with reduced zeugopod and limited movements at the elbow joint (Fig. 2C, D). There was complete agenesis of right thumb. Additionally, there was medial inclination of index finger and clinodactyly of 5^th^ finger. In the left hand, the thumb demonstrated minor sign of hypoplasia (Fig. 2C).

**Fig.3:**
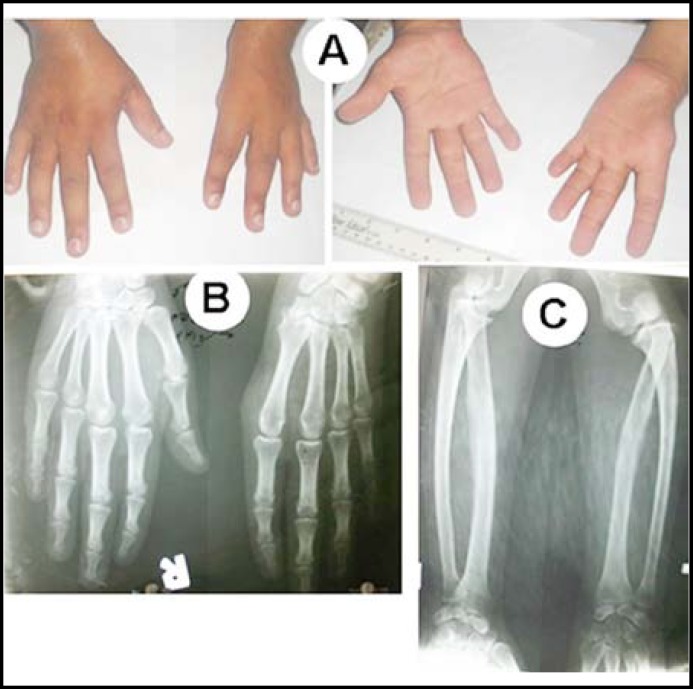
Phenotypic manifestation in patient VI

**Case VI: **The male subject was 26 years of age. There was unilateral aplasia of left thumb (Fig. 3A. The affected autopod had a reduced palm, while the wrist and elbow joints demonstrated limited extension/flexion movements. Additionally, there was shortening of the affected arm (Fig. 3A; Table-II). He was engaged in technical job and could manage his occupational duties mainly with the right hand. Radiographic study revealed absent trapezoid and hypoplastic scaphoid. There was fusion of trapezium and capitate, and lunate and triquertal (Fig. 3B). There was shortening of radius and ulna and the proximal head of radius was posteriorly dislocated (Fig. 3C). Fifth digits were short and demonstrated only one flexion crease, bilaterally. Radiographs of the right hand were unremarkable except crowding of carpals.

## DISCUSSION

We describe six independent patients with absent thumbs. According to the classification of thumb hypoplasia revised by Blauth and Schneider-Sickert^4^ and James *et al*.^5^, the phenotypes in all the cases were consistent with hypoplastic thumb “type-V”. The malformation was bilateral in two individuals (I, II), and unilateral in other four (III-VI). Interestingly, in the unilateral cases there was high preponderance of involvement of the left hand (n=3/4). Only in one case of unilateral thumb aplasia (i.e., patient V), there was minor hypoplasia of contralateral thumb. Other common observations in the affected autopods were clinodactyly or shortening of 5^th^ digits, short and narrow palms and reduced arm lengths. All the patients had normal IQ and there was no involvement of any other organ-systems. Family histories were devoid of thumb aplasia/hypoplasia in all cases. In patient IV however, there was occurrences of syndactyly and polydactyly in two different sibships.

The phenotypic spectrum of limb reduction defects in the Pakistani patients has not been well-described and only few cases have been reported.^9^^,^^10^ Malik and Jabeen reported a sporadic Pakistani case with thumb aplasia of right hand.^11^ That patient had additional findings of unilateral zygodactyly of left foot, low weight and a lean body. Another male patient, a product of consanguineous marriage, has been recently reported from Pakistan who had complete absence of thumb and index finger in left hand.^8^ The subject had additional findings of ulnar deficiency, shortening of left arm, reduced zeugopod and autopod, and severe flexion contracture at the elbow joint. In all of our present cases, there was isolated thumb deficiency. 

**Table-I:** Socio-demographic and biological attributes of patients with thumb aplasia

*Variable*	*Patient *					
	*I*	*II*	*III*	*VI*	*V*	*VI*
Gender (M, F)	F	M	M	F	M	M
Age (year)	45	6	6	3	7	26
Geographic origin (Pakistan)	South Punjab	Interior Sindh	South KPK	South KPK	North KPK	South KPK
Rural/Urban	R	R	U	U	R	R
Caste	Arain	Hindu/Bheel	Pathan	Pathan	Yousafzai	Mughal
Language/ethnicity	Punjabi	Marwari	Pashto	Pashto	Pashto	Pashto
Socio-economics	Low	Poor	Low-middle	Low-middle	Poor	Low
Family/house-hold type	Nuclear	Nuclear	Extended	Extended	Extended	Nuclear
Education/occupation	Nil/house wife	Nil	Student	Nil	Nil	Intermediate/ technical job
Marital status	Married	Single	Single	Single	Single	Single
Parental Consanguinity	FC	UR	UR	FC	UR	UR
Father age at patient’s birth (year)	23	34	26	27	35	39
Mother age at patient’s birth (year)	20	30	23	22	32	35
Patient’s parity	3 of 6	7 of 8	2 of 3	1 of 2	4 of 5	4 of 6
No. of normal sibs (B:S)	3:2	3:5	2:0	1:0	2:2	3:2
Family history of limb defect	No	No	No	Yes	No	No

FC= first cousins; KPK= Khyber Pakhtun-Khwa; UR= unrelated.

**Table-II:** Phenotypic manifestation of thumb aplasia in the recruited patients

**Phenotype **	Patient					
	I	II	III	VI	V	VI
Absent thumb	Both	Both	Left	Left	Right; left mild hypoplastic	Left
Index finger, medial inclination	R+, L++	R+, L+			R++	L+
Other digits	L. clinodactyly of 5^th^			5^th^ digit small	5^th^ clinodactyly	5^th^ digits small
Palm, thin/reduced	R+, L+	L+	L+	L+	R+	L+
Arm, reduced/short	R+, L+	R+, L+	L+	L++	R++	L+

L= left; R= right; + = mild; ++ = severe

**(A—C): **Photographs and radiographs of patient I.

**(D):** Photograph of patient II. **(E):** Photograph of patient III.

**(A): **Pedigree of patient IV, here symbolized as filled circle in generation III (III-2). Family members II-5 and II-6 had polydactyly and syndactyly, respectively.

**(B). **Photograph of patient IV. **(C-D): **Photograph of patient V.

**(A—C): **Photographs and radiographs.

Thumb is responsible for proper functioning of hand that is important for independent life activities. Thumb aplasia is usually associated with reduction of hand musculature, narrowing of palm and reduction the size of arm/hand, which negatively affects functioning of hand and in some cases cause great psychological burden for the child/patient. Thus, the parents have responsibility of helping their children in dealing with their feelings of grief, responsibility, and guilt.^12^ In our study, it was observed that the individuals had adopted the situations accordingly. For instance, patient I was well-trained to perform almost all her house-hold activities. However, she had difficulty in carrying heavy articles and fast-grasping of objects. Patients with thumb agenesis develop the habit of grasping objects between the index and long fingers. In the situations with unilateral thumb agenesis, the patient remained more dependent on the contralateral normal hand.

The molecular etiology of thumb hypoplasia/aplasia remains less described.^5^ There is no specific genetic factor known to cause isolated thumb aplasia. However, genetic basis of several syndromic form of first digital ray deficiency have been discovered. Holt-Oram syndrome (OMIM-142900) involves absent thumbs along with radial deficiency and cardiac anomalies.^1^^,^^2^ One form of Holt-Oram syndrome is caused by mutations in *TBX5*. Fanconi anemia (OMIM-227650) is characterized by radial deficiency in addition to leukemia and symptoms in kidneys and skin, and is known to be caused by at least thirteen different genes.^1^ Similarly, Duane-radial ray syndrome (OMIM-607323) is similar to Holt-Oram syndrome with additional symptoms in eyes, ears and kidneys, and is associated with mutations in *SALL4*. VACTERAL syndrome which is characterized by absent or hypoplastic thumb along with complications in vertebral, renal, cardiac and respiratory systems is known to be caused by mutations in *TCSK5*.^1^^,^^2^

Thumb is a vital digit responsible for proper functioning of hand. For the management of thumb agenesis, pollicisation of the index finger is generally performed in which the index finger is placed at the position of thumb. In this case, the subject is able to perform normal functions with three fingers and one thumb.^13^ Majority of the Pakistani subjects with limb deficiencies are less fortunate in finding expert surgical opinion to manage their conditions. In most of the situations, the affected individuals belong to low socio-economic background and are unable to bear the expenses of surgical procedures. Even though all of our recruited subjects with thumb aplasia otherwise had normal lives and had adopted to perform their daily activities with the existing four fingers in the involved hands; however, pollicisation procedures of the affected hands of these individuals could be highly beneficial for their occupational and personal lives, and would remarkably improve their standards of living. Thumb agenesis is uncommon, and most of the isolated cases are sporadic and non-familial, thus limiting the number of patients who are available for molecular analyses. Therefore, collection of such cases is imperative for successful molecular genetics investigations. Recent advances in technology like comparative genomic hybridization, SNP arrays and whole-exom analyses could help in the identification of causative mutations in sporadic cases.
